# Supplementary figures and images for: Spatiotemporal co-distribution and time lagged cross correlation of malaria and dengue in Loreto, Peru
Source: PLOS Glob Public Health. 2025 Dec 4;5(12):e0005598. doi: 10.1371/journal.pgph.0005598 (PMC12677548; doi:10.1371/journal.pgph.0005598)

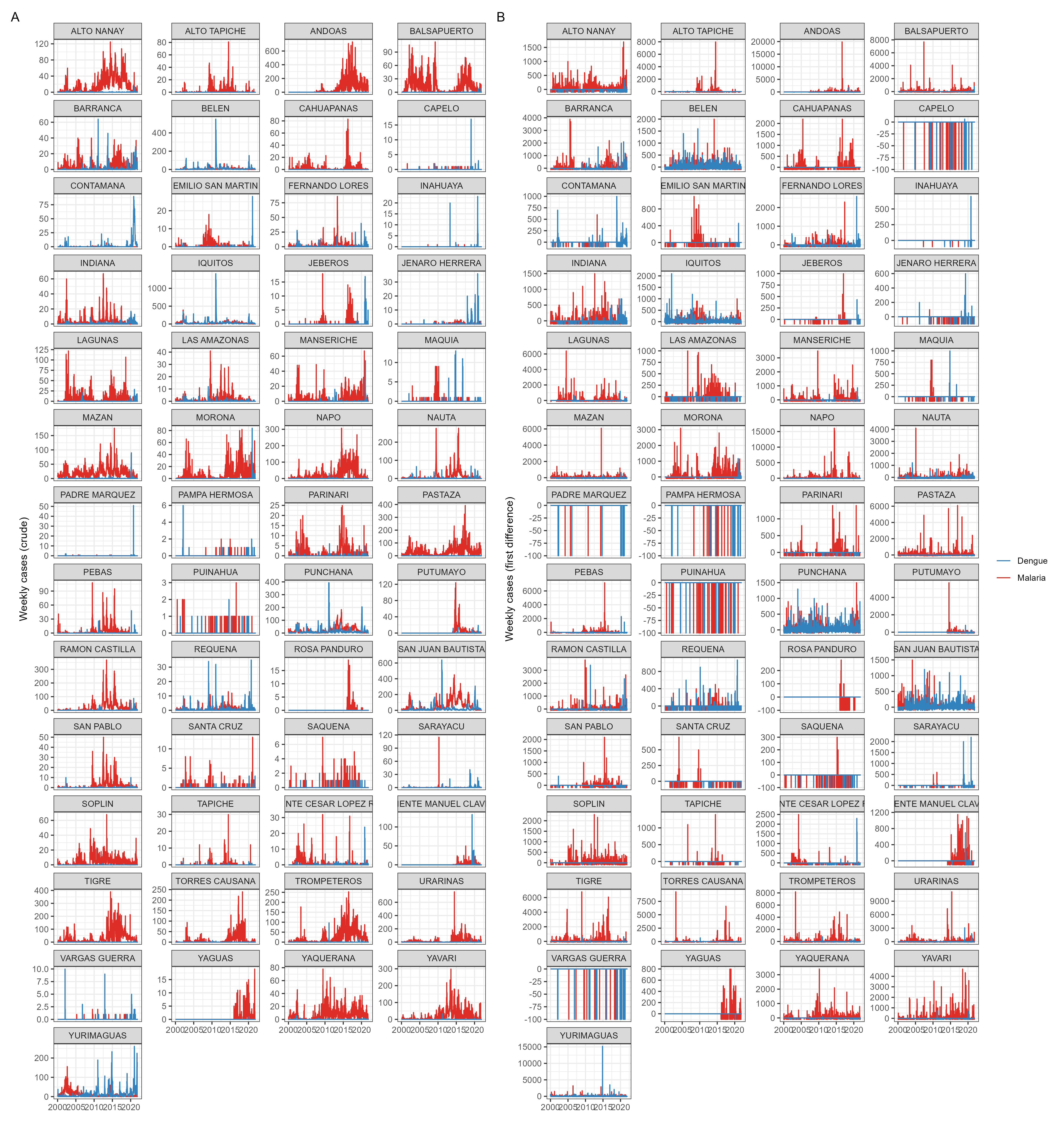

Supplement: S1 Fig — (A) Crude weekly cases. (B) First difference of weekly cases. (PNG) [file pgph.0005598.s001.png]

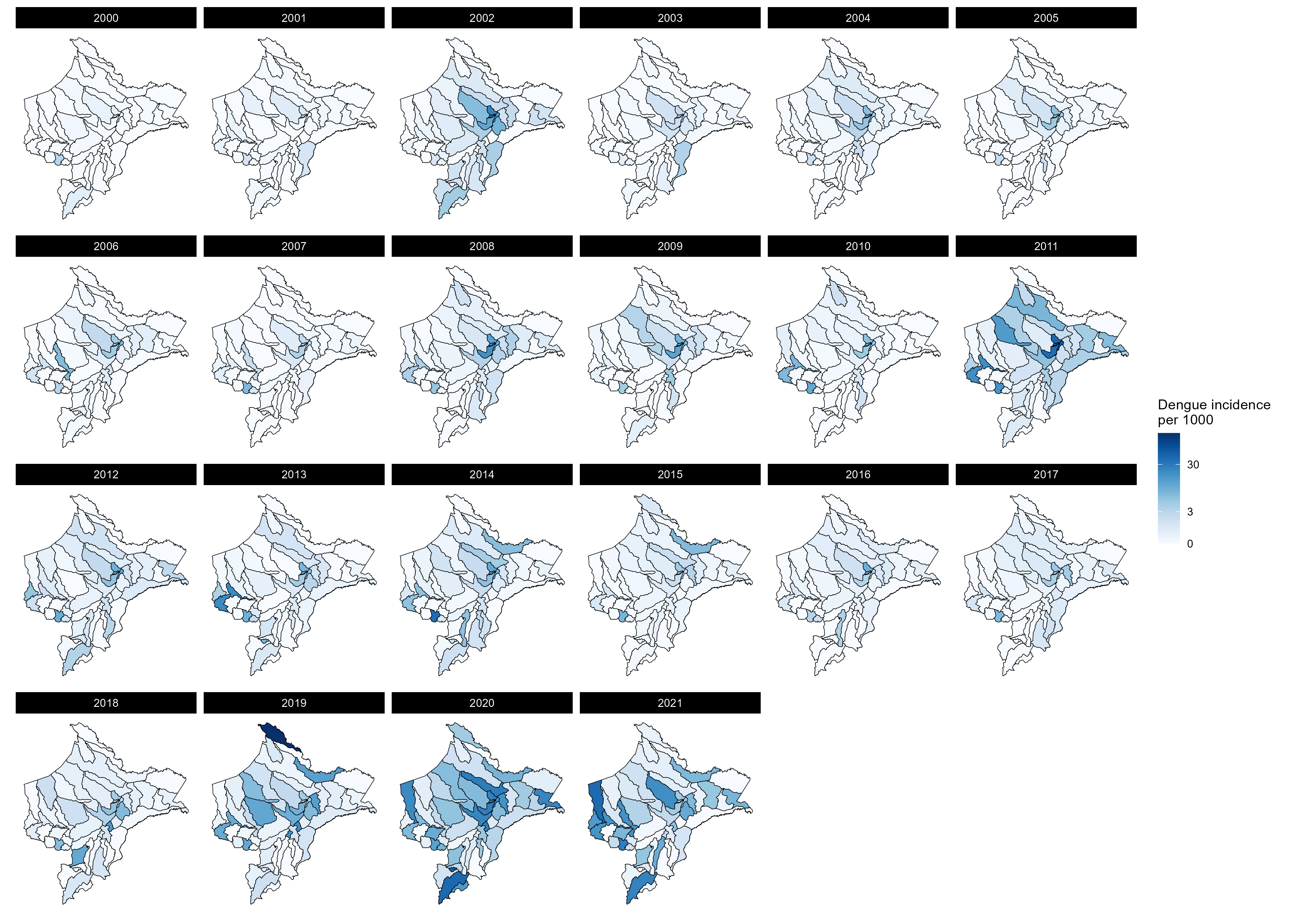

Supplement: S2 Fig — Maps produced in R v.4.5 using public data from Instituto Nacional de Estadística e Informática (INEI - Peru) contributors (https://estadist.inei.gob.pe/map) under Open Data Commons Open Database License (ODbL) 1.0 (http://openstreetmap.org/copyright). (PNG) [file pgph.0005598.s002.png]

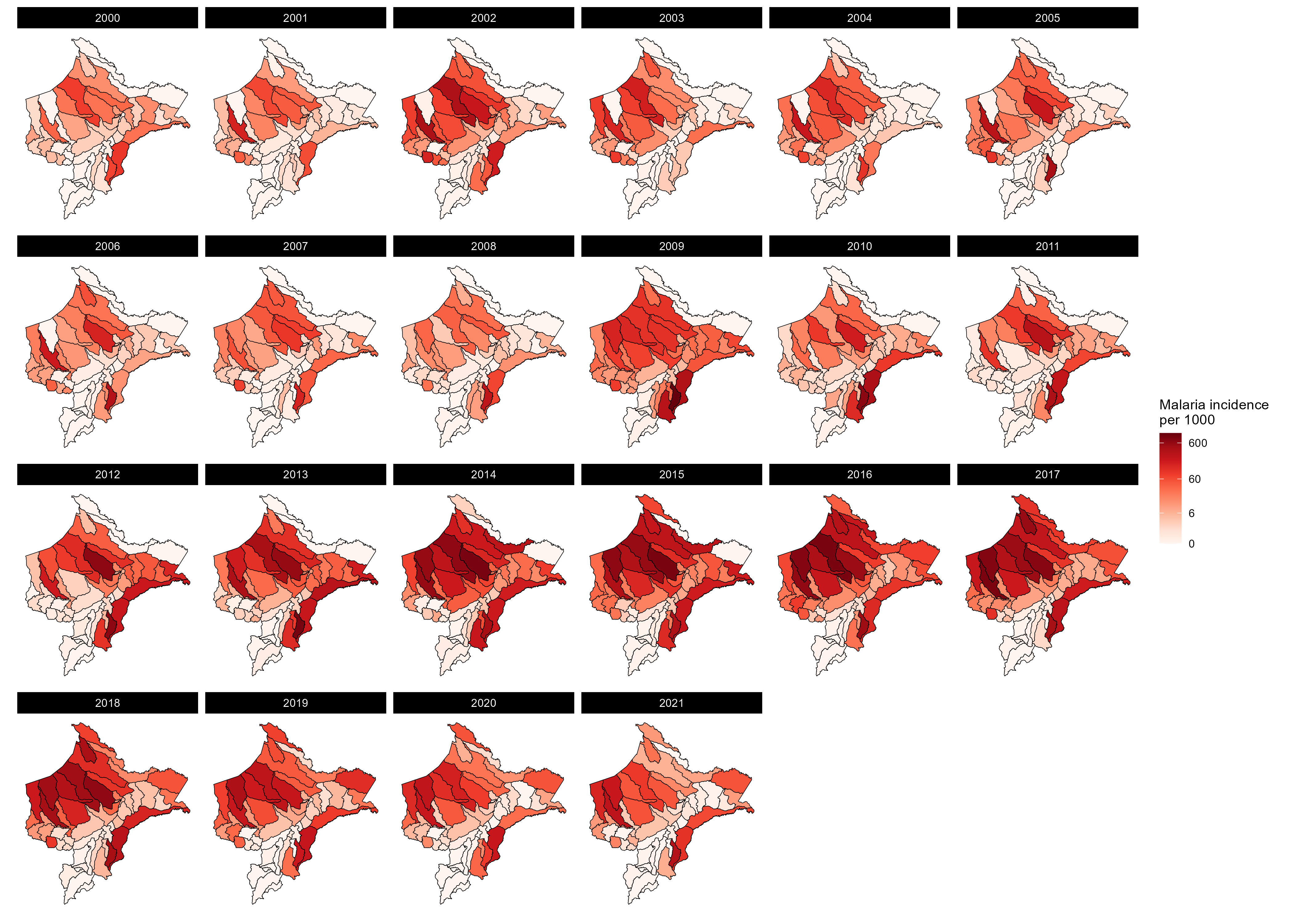

Supplement: S3 Fig — Maps produced in R v.4.5 using public data from Instituto Nacional de Estadística e Informática (INEI - Peru) contributors (https://estadist.inei.gob.pe/map) under Open Data Commons Open Database License (ODbL) 1.0 (http://openstreetmap.org/copyright). (PNG) [file pgph.0005598.s003.png]

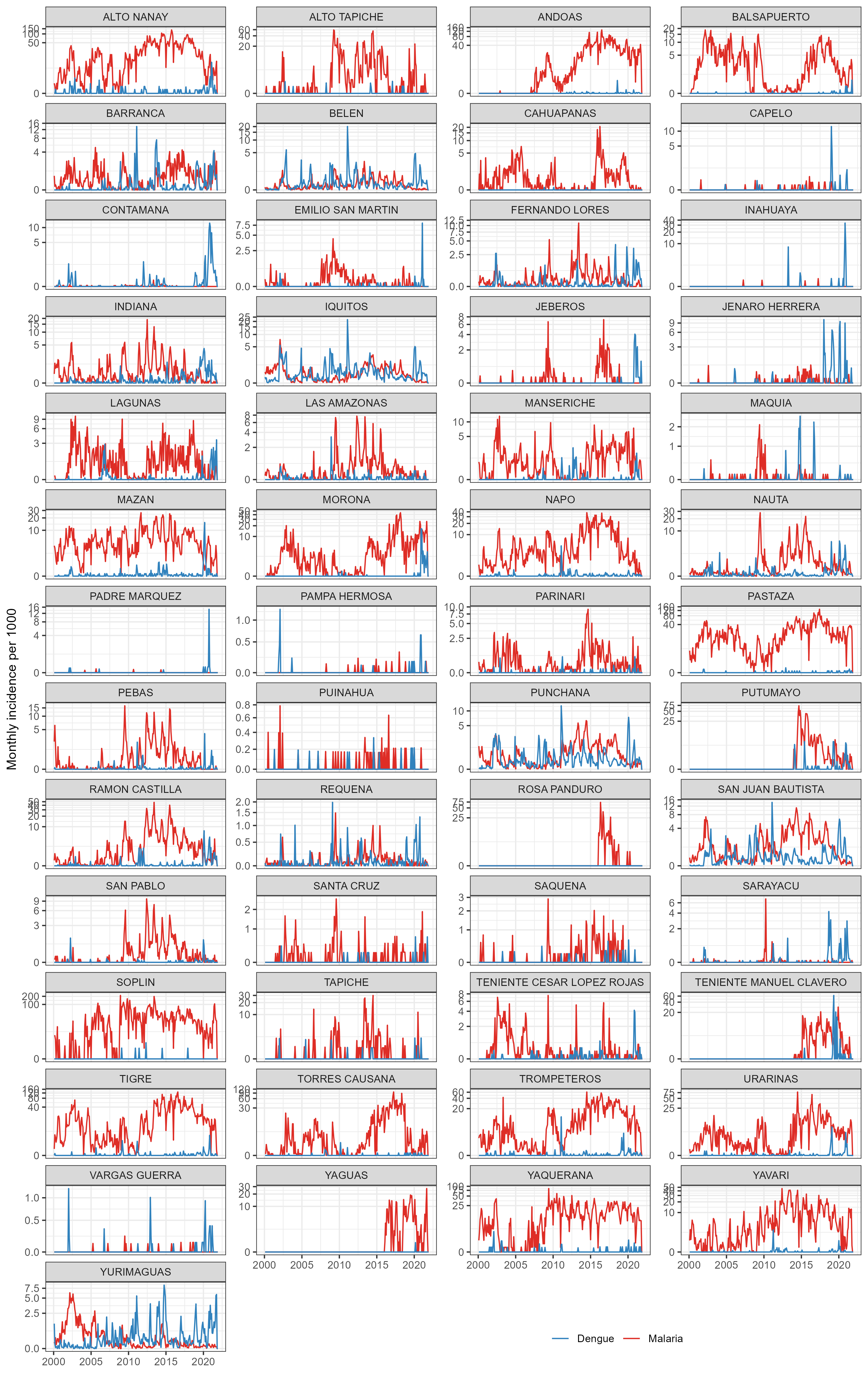

Supplement: S4 Fig — (PNG) [file pgph.0005598.s004.png]

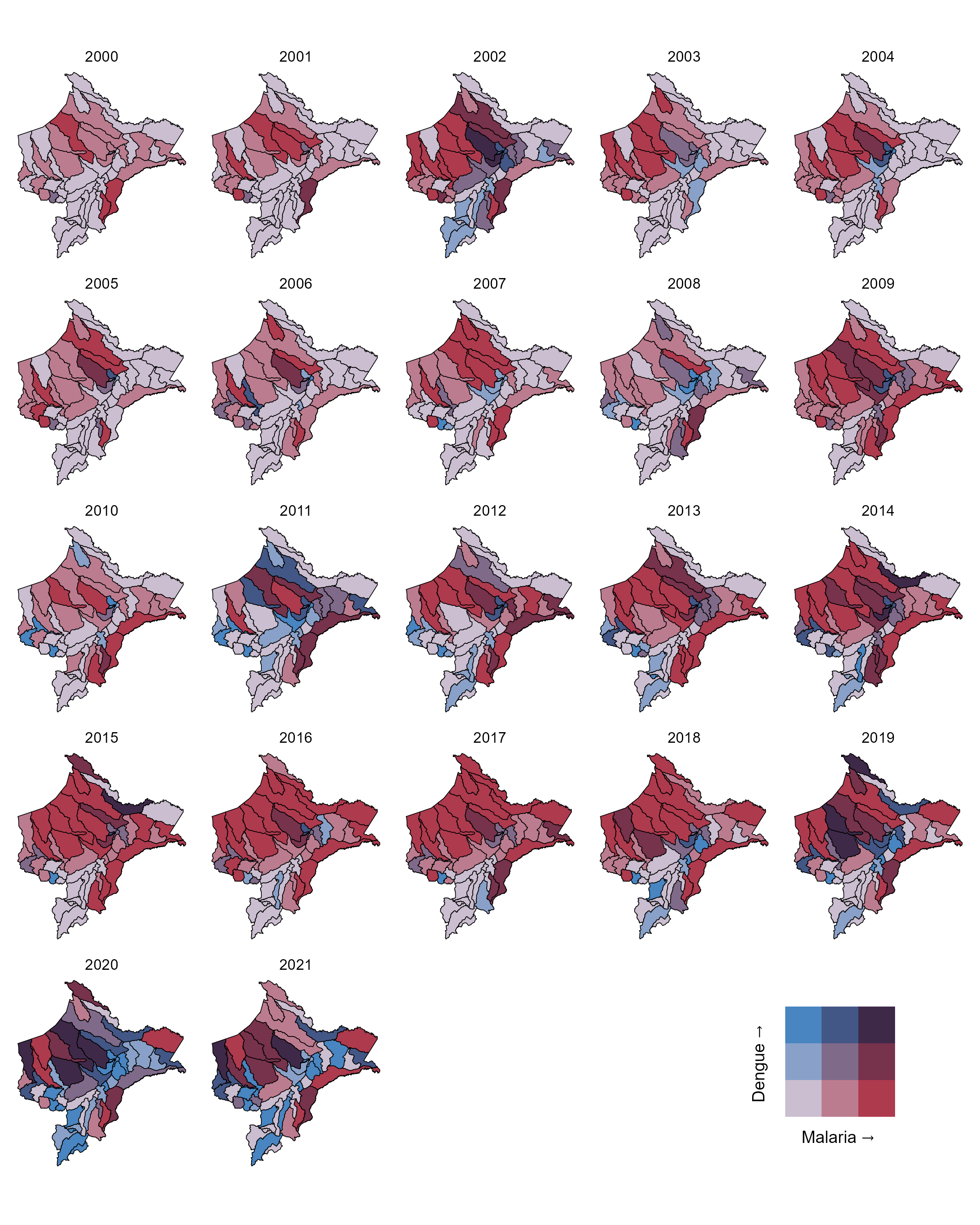

Supplement: S5 Fig — Dengue and malaria log-incidence (categorised using Fisher-Jenks algorithm for the entire study period). Maps produced in R v.4.5 using public data from Instituto Nacional de Estadística e Informática (INEI - Peru) contributors (https://estadist.inei.gob.pe/map) under Open Data Commons Open Database License (ODbL) 1.0 (http://openstreetmap.org/copyright). (PNG) [file pgph.0005598.s005.png]

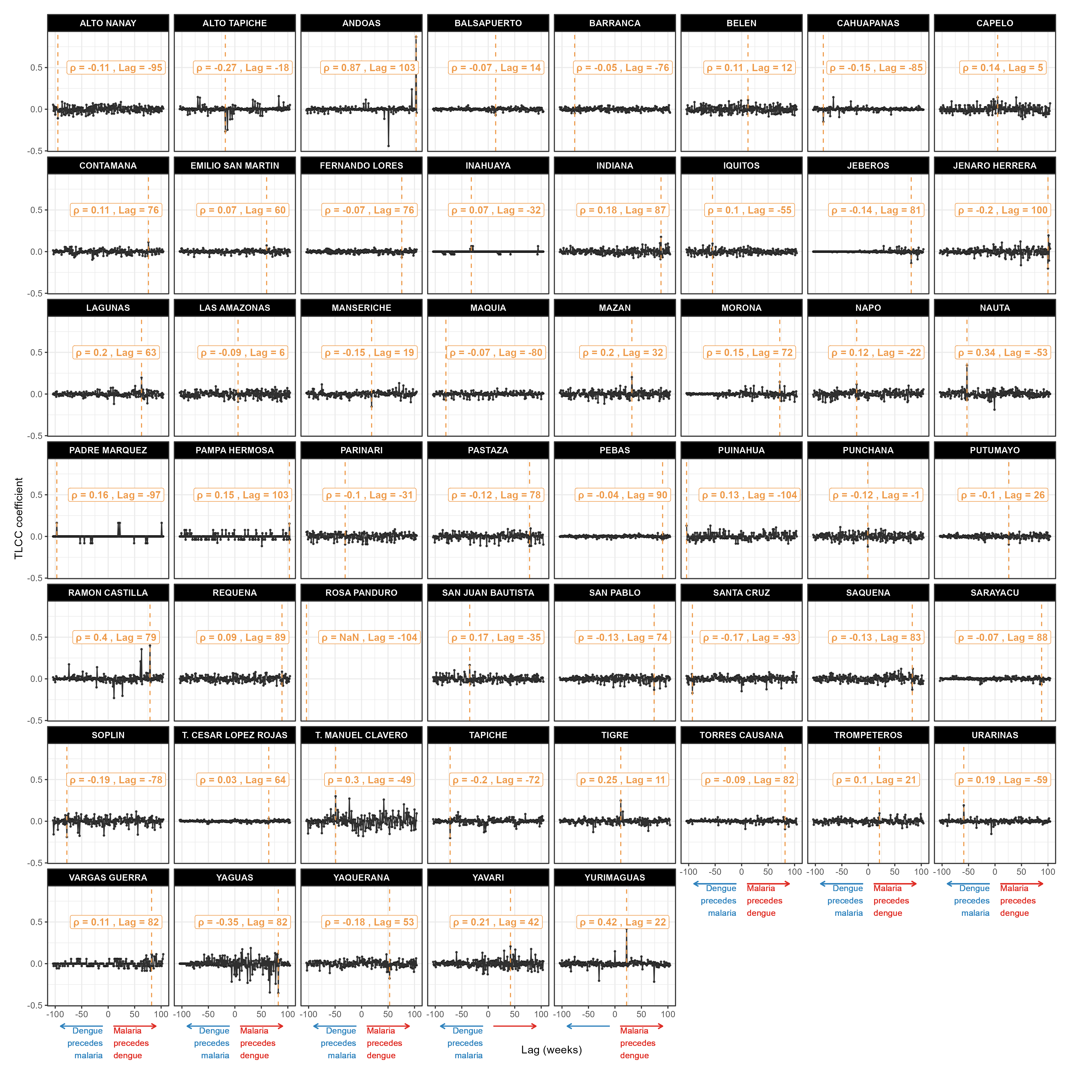

Supplement: S6 Fig — ρ indicates the maximum correlation coefficient and lags are in weeks. (PNG) [file pgph.0005598.s006.png]

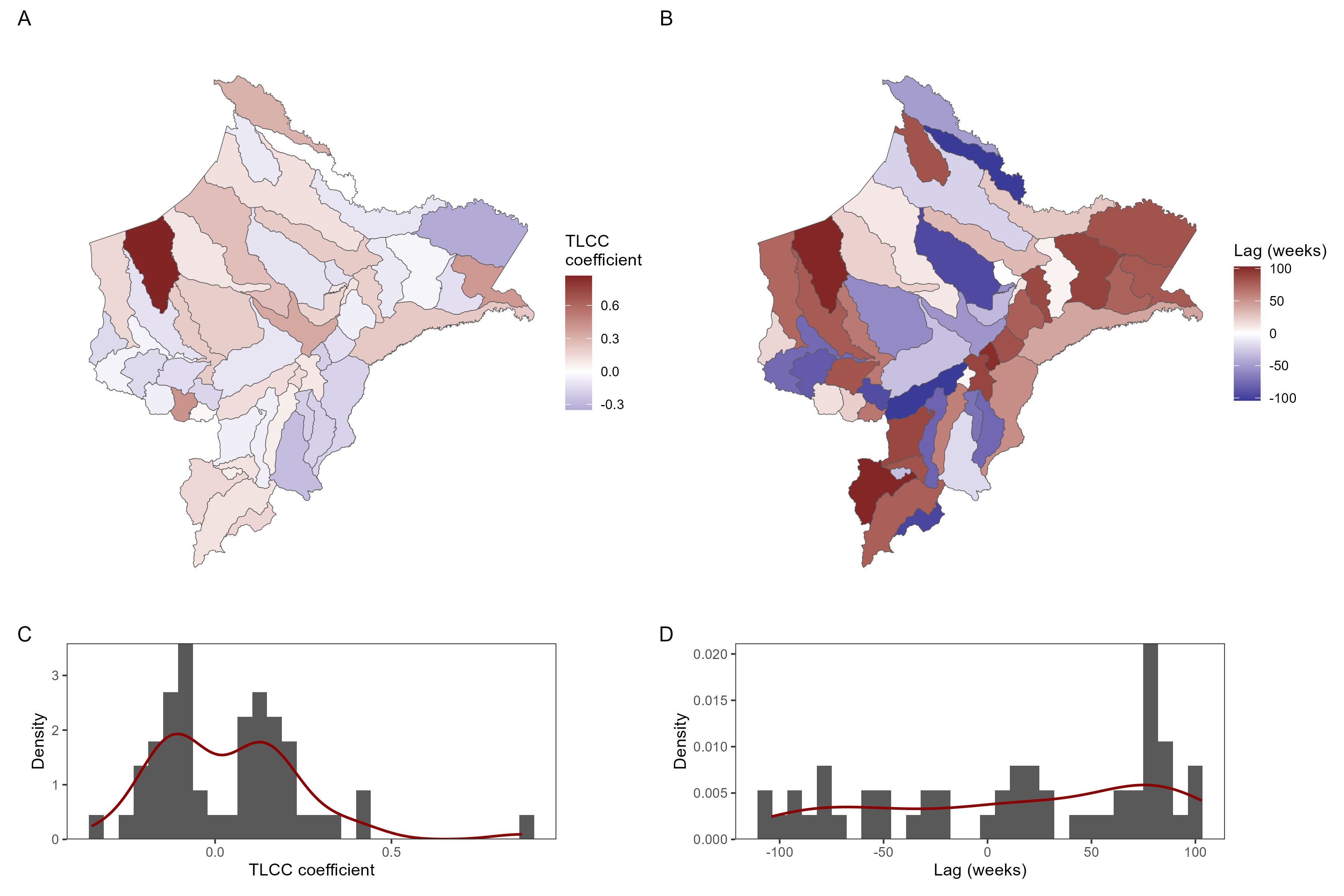

Supplement: S7 Fig — (A) TLCC coefficient by district (highest absolute value plotted). (B) Lag time in weeks for maximum TLCC coefficient by district. Maps produced in R v.4.5 using public data from Instituto Nacional de Estadística e Informática (INEI - Peru) contributors (https://estadist.inei.gob.pe/map) under Open Data Commons Open Database License (ODbL) 1.0 (http://openstreetmap.org/copyright). (PNG) [file pgph.0005598.s007.png]

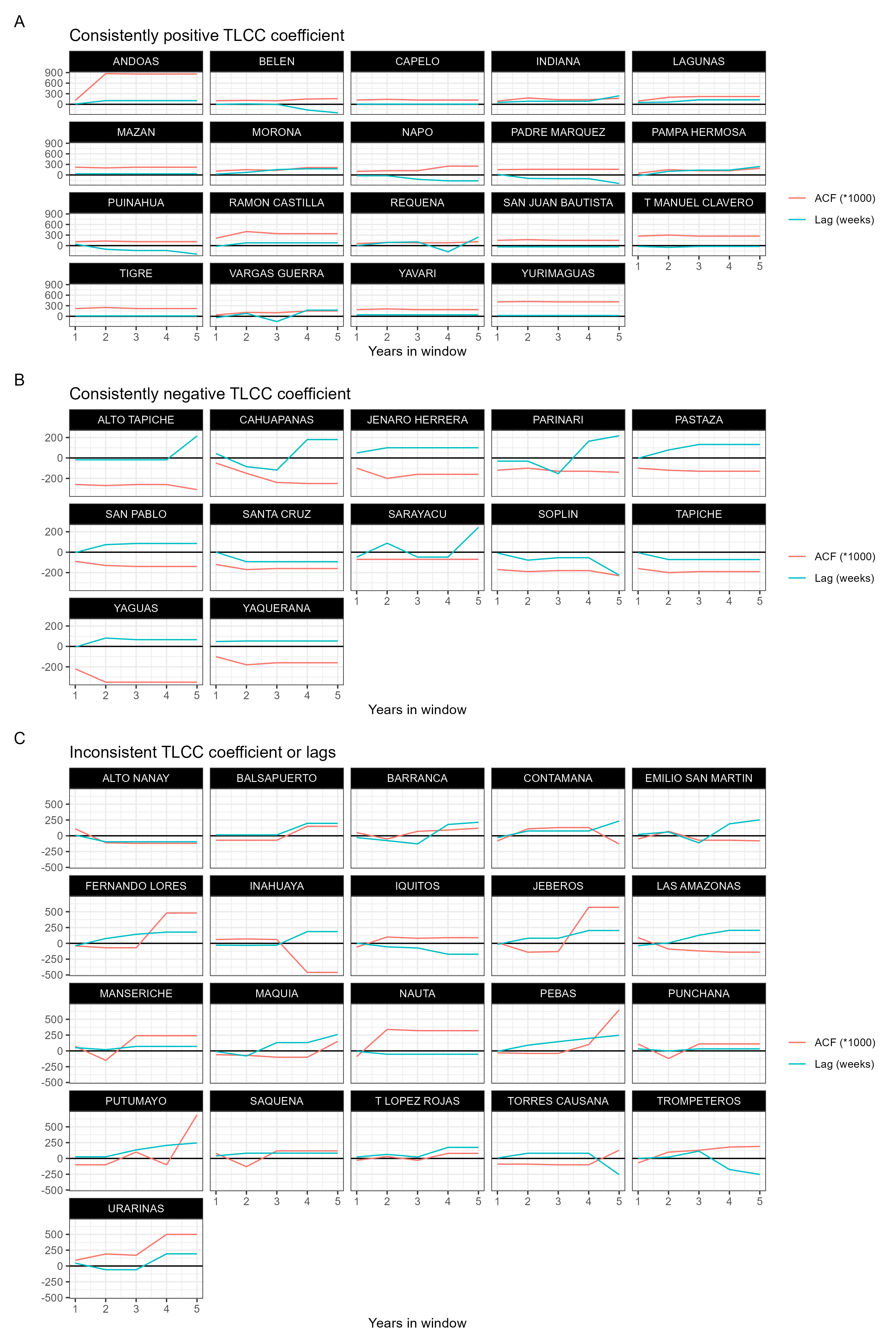

Supplement: S8 Fig — TLCC analysis was repeated using time windows of 1–5 years. The maximum TLCC coefficient (scaled times 1000) and corresponding lag for each time window and district are plotted. Districts are categorised qualitatively into those with consistently positive coefficients (A), those with consistently negative coefficients (B), and those with inconsistent TLCCs or lags (C). (PNG) [file pgph.0005598.s008.png]

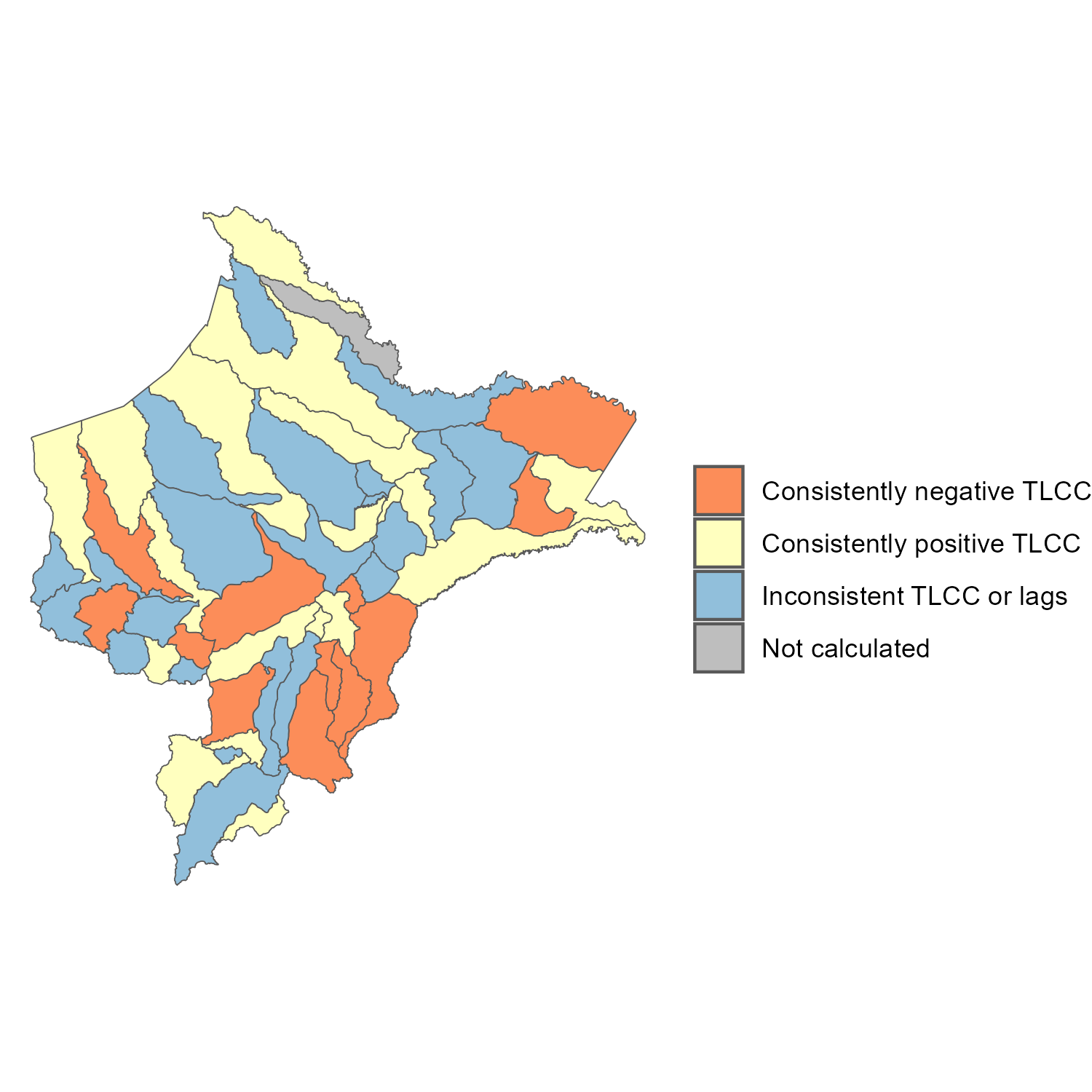

Supplement: S9 Fig — Maps produced in R v.4.5 using public data from Instituto Nacional de Estadística e Informática (INEI - Peru) contributors (https://estadist.inei.gob.pe/map) under Open Data Commons Open Database License (ODbL) 1.0 (http://openstreetmap.org/copyright). (PNG) [file pgph.0005598.s009.png]

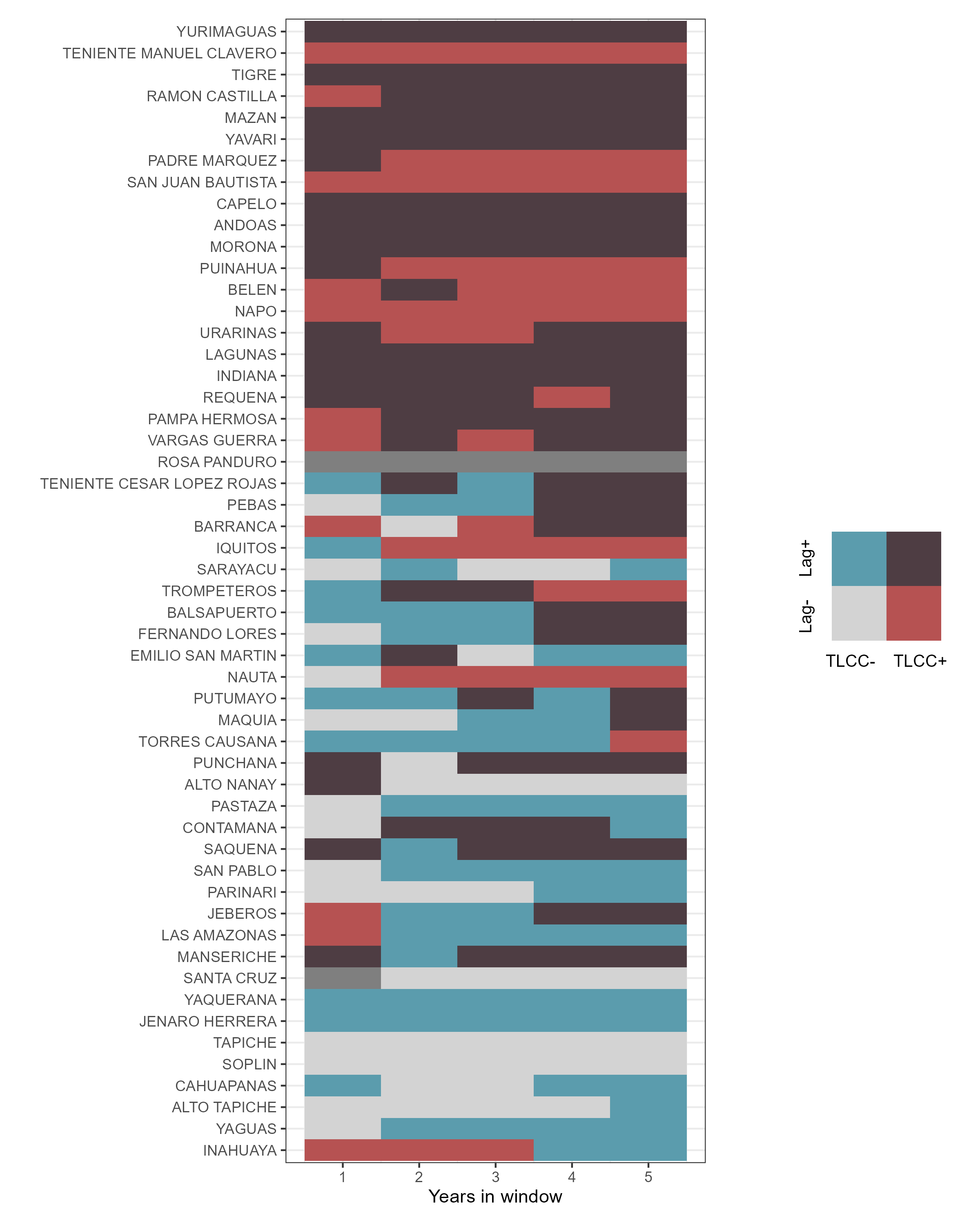

Supplement: S10 Fig — Districts are ordered from highest to lowest TLCC coefficient in any of the five tested windows. Purple tiles had a positive TLCC coefficient and positive corresponding lag. Pink tiles had a positive TLCC coefficient and negative corresponding lag. Light blue tiles had a negative TLCC coefficient and positive corresponding lag. Light grey tiles had a negative TLCC coefficient and a negative corresponding lag. (PNG) [file pgph.0005598.s010.png]
