# Supplementary material for: Spatiotemporal co-distribution and time lagged cross correlation of malaria and dengue in Loreto, Peru
Source: PLOS Glob Public Health. 2025 Dec 4;5(12):e0005598. doi: 10.1371/journal.pgph.0005598 (PMC12677548; doi:10.1371/journal.pgph.0005598)
Supplement: Attachment — Submitted filename: 2025_09_27 rebuttal letter.docx [file pgph.0005598.s011.docx]

September 26, 2025

Dear reviewers,

We thank you for the comments and revisions to help enhance our manuscript. I am re-submitting, on behalf of all authors, the attached revised manuscript, titled “Spatiotemporal co-distribution and time lagged cross correlation of malaria and dengue in Loreto, Peru”. Please find an itemized list of our responses below.

Journal Requirements:

1. Please provide additional details regarding participant consent. In the ethics statement in the Methods and online submission information, please ensure that you have specified (1) whether consent was informed and (2) what type you obtained (for instance, written or verbal, and if verbal, how it was documented and witnessed). If your study included minors, state whether you obtained consent from parents or guardians. If the need for consent was waived by the ethics committee, please include this information.

*We have modified the ethics statement to explicitly state that the requirement for consent was waived because data was de-identified and collected as part of routine public health surveillance.*

2. If you are reporting a retrospective study of medical records or archived samples, please ensure that you have discussed whether all data were fully anonymized before you accessed them and/or whether the IRB or ethics committee waived the requirement for informed consent. If patients provided informed written consent to have data from their medical records used in research, please include this information.

   *The ethics statement explicitly states that the data were fully anonymized and the requirement for consent was waived.*
3. Figure 1 and 3: please (a) provide a direct link to the base layer of the map (i.e., the country or region border shape) and ensure this is also included in the figure legend; and (b) provide a link to the terms of use / license information for the base layer image or shapefile. We cannot publish proprietary or copyrighted maps (e.g. Google Maps, Mapquest) and the terms of use for your map base layer must be compatible with our CC-BY 4.0 license.

   *We have included information on the source of the base layer for the map (*[*https://estadist.inei.gob.pe/map*](https://estadist.inei.gob.pe/map)*) and terms of use (Open Data Commons Open Database License) in the legends for figures 1 and 3.*
4. The work is interesting, but it would be important to make the methods used more understandable by giving more suitable details, as suggested by reviewer 2. There is also the question of data origin and availability (site Regional Health Department of Loreto?)

   *We have addressed all suggestions made by Reviewer 2, as detailed below, and clarified the origin of the data in the “Data sources” section.*

Reviewers' comments:

Reviewer #1:

1. The manuscript was done correctly and the data supports the results presented and the highly relevant public health findings, however, I have a question:
   Is the data used in the analysis from a public database? I couldn't find their source anywhere in the manuscript. I would suggest adding "Data sources" before "Study area" to provide greater clarity as to where the data were obtained.

   *We have included clarifications in the “Data sources” section in the Methods. The data were obtained from the Loreto Regional Health Department through an official request. The dataset is not currently directly downloadable but can be requested through Peru’s public information access system (https://www.gob.pe/20399-solicitar-acceso-a-la-informacion-publica).*
2. Include the R packages used to construct the maps, as they may be subject to copyright.
    *Shapefiles to create the maps were obtained from the Instituto Nacional de Estadística e Informática (INEI Peru, https://estadist.inei.gob.pe/map). We have included information on the source of the base layer for the map and terms of use (Open Data Commons Open Database License) in the legends for figures 1 and 3.*
3. In Figure 2, the titles should be better organized, as the position of "Lag (weeks)" is confusing to the reader.

   *Thank you for the suggestion. We have rearranged the x-axis labels in Figure 2 to enhance readability and interpretability.*
4. In sentences 274 to 277, the authors could better explain the environmental and geographic characteristics of the area and their associations.

   *We have edited the discussion to better explain that small-scale differences in environmental and geographic characteristics as well as land use could explain the differences in observed associations. We explain how different stages of deforestation can lead to differential effects for the vectors of malaria and dengue and consequently differential exposures and disease risk, as well as how protected areas can limit land conversion and affect observed spatial patterns.=.*

Reviewer #2:

1. The manuscript would benefit from a clearer statement of the research question and motivation. Why is it important to investigate time-lagged correlations between these diseases? What hypotheses are being tested? Clarifying the added value of this approach would help frame the relevance of the work.

   *We have reworked the last paragraphs of the introduction to make our research question, hypothesis, and the added value of our approach more clear.*
2. The Abstract should explicitly state that the study compares dengue and malaria incidence.

   *We have modified the abstract to reflect that the study compares dengue and malaria incidence.*
3. The sentence “The number of districts reporting both diseases has increased” appears abruptly. Consider a more informative formulation, e.g., “Our analysis shows a growing number of districts reporting both dengue and malaria cases over time.”

   *Thank you for the suggestion, we have incorporated the suggested formulation into the abstract.*
4. Some phrases are overly technical for the Abstract, such as “We categorised districts based on dengue/malaria spatio-temporal patterns and conducted Moran’s tests...” A more accessible formulation could be: “We grouped districts based on shared temporal and spatial patterns in dengue and malaria incidence and explored how the two diseases co-occurred geographically.”

   Thank you for this suggestion. We have incorporated the suggested wording into the abstract.
5. Line 66: “While these two VBD share many similarities” appears disconnected. The diseases should be clearly introduced earlier to give context.

   *We have moved this paragraph so it appears after malaria and dengue in Peru are introduced.*
6. Line 99: The authors could be more precise and state that the study examines the incidence of the two diseases.

   *We have edited this sentence to explicitly state that we are examining the incidence of dengue and malaria.*
7. The Methods mention weekly incidence, but Figure 1 presents monthly case counts. Please clarify how the monthly incidence was calculated.

   *We have modified subsection “Data sources” to clarify that raw data included individual case observations with date of symptom onset. Data was aggregated by month of onset for visualization in figure 1, and by week of onset for the primary analysis.*
8. Figure 1 should be moved to the Results section, as it presents outputs from the descriptive analysis rather than methodological diagrams.

   *We have moved Figure 1 to the Results section.*
9. Consider adding disaggregated incidence plots by district. These could reveal meaningful trends and enhance the interpretation of the district-level analyses.

   *We have added disaggregated incidence plots by district as supplementary figure S3.*
10. Line 152 states that first differences were applied to ensure stationarity. It would be important to demonstrate that this step is sufficient (e.g., via the Augmented Dickey-Fuller test). Consider including supplementary plots of the differenced series for transparency.

    *Thank you for the suggestion. We have conducted Augmented Dickey-Fuller tests on all the district-level case series after first differences were applied, and p-values were all <0.01, indicating that case series were stationary. We have added text in the “Statistical analyses” subsection of the methods to clarify this, as well as included code to conduct this analysis in the GitHub repository.*
11. Please expand the Methods to include information on the significance thresholds used, the criteria for determining optimal lags, and how the Fisher-Jenks algorithm was applied.

    *We have included information on the significance threshold we used (α = 0.05), the criteria for determining optimal lags (the maximum absolute cross-correlation coefficient for each district) and how the Fisher-Jenks algorithm was applied (using the pooled distribution of incidence for each disease over the entire study period, with three classes, using the cartography package in R).*
12. Critically, the manuscript should describe how seasonal patterns were handled. Seasonal differencing is essential when performing time-lagged cross-correlation on seasonal time series, to avoid spurious associations driven by periodic fluctuations.

    *Thank you for pointing this out. We have added a section describing how seasonality was tested for and handled (lag-52 difference to each weekly time series) and adjusted the results accordingly. Some of the values have changes slightly with this adjustment, but overall trends and conclusions remain the same.*
13. Clarify how districts in Figure 2 were selected—was this based on significance, magnitude of correlation, or another criterion?

    *We have clarified in the legend for Figure 2 that the districts with the highest coefficient in each of the four presented groups were selected for this illustration.*
14. The conclusion that this framework could inform integrated control strategies is intriguing, but needs to be substantiated. The authors should specify what type of strategies could be informed and how, based on the current analysis. Are there operational implications for timing interventions or resource allocation?

    *Thank you for this observation. We have added a paragraph to the discussion specifying potential strategies that could be integrated and how their timing could be informed by this analysis.*
15. The GitHub repository is a valuable resource. It would be helpful to provide a short summary or guide in the README file outlining the structure of the code and how users can reproduce key analyses.

    *We have updated the README file to include the structure of the repository and instructions to reproduce analyses included in the manuscript (https://github.com/healthinnovation/loreto_synchrony).*
